# Fluid intelligence and brain functional organization in aging yoga and meditation practitioners

**DOI:** 10.3389/fnagi.2014.00076

**Published:** 2014-04-22

**Authors:** Tim Gard, Maxime Taquet, Rohan Dixit, Britta K. Hölzel, Yves-Alexandre de Montjoye, Narayan Brach, David H. Salat, Bradford C. Dickerson, Jeremy R. Gray, Sara W. Lazar

**Affiliations:** ^1^Massachusetts General Hospital, Harvard Medical SchoolCharlestown, Boston, MA, USA; ^2^Bender Institute of Neuroimaging, Justus Liebig Universität GiessenGiessen, Germany; ^3^Faculty of Psychology and Neuroscience, Maastricht UniversityMaastricht, Netherlands; ^4^Information and Communication Technologies, Electronics and Applied Mathematics Institute, Université Catholique de LouvainLouvain-La-Neuve, Belgium; ^5^BrainBotSan Francisco, CA, USA; ^6^Institut für Medizinische Psychologie, Charite UniversitätsmedizinBerlin, Germany; ^7^Media Lab, Massachusetts Institute of TechnologyCambridge, MA, USA; ^8^PGSP-Stanford Psy.D. ConsortiumPalo Alto, CA, USA; ^9^VA Boston Healthcare SystemBoston, MA, USA; ^10^Department of Psychology, Michigan State UniversityEast Lansing, MI, USA

**Keywords:** aging, fluid intelligence, graph theory, brain network integration, brain network segregation, brain network resilience, mindfulness, yoga

## Abstract

Numerous studies have documented the normal age-related decline of neural structure, function, and cognitive performance. Preliminary evidence suggests that meditation may reduce decline in specific cognitive domains and in brain structure. Here we extended this research by investigating the relation between age and fluid intelligence and resting state brain functional network architecture using graph theory, in middle-aged yoga and meditation practitioners, and matched controls. Fluid intelligence declined slower in yoga practitioners and meditators combined than in controls. Resting state functional networks of yoga practitioners and meditators combined were more integrated and more resilient to damage than those of controls. Furthermore, mindfulness was positively correlated with fluid intelligence, resilience, and global network efficiency. These findings reveal the possibility to increase resilience and to slow the decline of fluid intelligence and brain functional architecture and suggest that mindfulness plays a mechanistic role in this preservation.

## Introduction

Neural structure, function, and cognitive performance naturally decline as individuals age (Morrison and Hof, 1997; Salat et al., 2004; Persson et al., 2006; De Chastelaine et al., 2011). However, research suggests that a variety of cognitive training programs (Nyberg et al., 2003; Willis et al., 2006; Belleville et al., 2011; Anguera et al., 2013) and aerobic exercise (Colcombe et al., 2004; Holzschneider et al., 2012) can improve cognitive performance and associated brain function in older adults. This suggests that older adults can still undergo beneficial neuroplastic changes through changes in behavior. Besides specific cognitive abilities, even the higher level construct of fluid intelligence, i.e., the set of abilities involved in coping with novel environments and abstract reasoning (Sternberg, 2008), has recently been shown to be trainable through working memory training (Jaeggi et al., 2008), and related to changes in brain function (Jausovec and Jausovec, 2012). However, generalizability of working memory training is not unequivocal (Melby-Lervag and Hulme, 2013) and so far training-induced improvements in fluid intelligence and associated brain function have been demonstrated only in young people. Their effects on older individuals remain to be investigated.

There is growing evidence that meditation could potentially reduce age-related decline in cognition and brain function (for a review see Gard et al., 2014). For example, older meditators have been shown to outperform age-matched participants on an attentional blink task (Van Leeuwen et al., 2009) and on tasks assessing attention, short-term memory, perceptual speed, and executive functioning (Prakash et al., 2012). Pagnoni and Cekic (2007) reported that the typical negative correlation between age and sustained attention was not present in Zen meditators. At the neural level, two studies found negative correlations between age and gray matter volume in controls, but not in age-matched meditation practitioners (Lazar et al., 2005; Pagnoni and Cekic, 2007). Another study found a less prominent age-related decline in fractional anisotropy in multiple white matter tracts in meditators compared to controls (Luders et al., 2011). These findings suggest that meditation practice may be able to reduce normal age-related cognitive decline and neuro-degeneration. However, a limitation of these studies is that, although groups were matched for sex, age, and education, other important factors including exercise and cognitive engagement (Kramer and Erickson, 2007; Plassman et al., 2010; Fotuhi et al., 2012; Wilson et al., 2012) were not controlled for. Furthermore, these studies focused on specific cognitive functions, mostly attention, as opposed to higher level constructs such as fluid intelligence, which could have broader implications due to its high predictive value of real life behavior (Gray and Thompson, 2004; Deary, 2012).

There is growing evidence that yoga can also enhance cognition (Chattha et al., 2008; Subramanya and Telles, 2009; Kyizom et al., 2010; Rocha et al., 2012) and brain function (Streeter et al., 2010) in younger adults. With only one publication, the literature regarding the effects of yoga on cognition in older people is sparse and equivocal (Oken et al., 2006). The various yoga and meditation traditions share some similarities, but differ in practices as well as theoretical orientation (Goleman, 1996). Few scientific studies have directly compared practitioners of different traditions to determine similarities and differences in either the benefits or mechanisms underlying improved cognition or health.

To investigate the impact of yoga and meditation practices on brain function, we have employed graph theoretical methods to assess group differences in resting state brain connectivity. These graph methods have been successfully used to reveal a negative relation between age and network integration (Achard and Bullmore, 2007), and a positive one between fluid intelligence and both network integration and segregation (Van Den Heuvel et al., 2009; Langer et al., 2012). However, the effects of training on functional brain network architecture have not yet been studied in the context of aging.

Graph-based analysis also provides novel ways to simulate the effect of aging-related brain damage, and the brain's resilience to this damage (Achard et al., 2006). Resilience analysis has been used to simulate neuronal death (Rubinov et al., 2009), and it has been speculated that the dynamic impact of brain lesions is related to cognition and behavior (Alstott et al., 2009). While structural brain networks of Alzheimer's patients have been shown to be less resilient against targeted attacks than networks of healthy controls (He et al., 2008), network resilience has not yet been assessed in populations that display above average cognitive function at older age.

Here we used graph theoretical methods to investigate the effect of age on resting state functional brain connectivity and fluid intelligence in yoga and meditation practitioners, and in controls. The three groups were demographically well matched and we controlled for variables that might influence age-related decline. We hypothesized that yoga and meditation practitioners would be less subject to age-related declines in fluid intelligence and the resting state network properties of small-worldness and integration. No age-related decline in network segregation was expected in any of the groups (Meunier et al., 2009). Furthermore, we investigated differences in the resilience of brain functional networks between the three groups. Finally, we assessed the relationship between mindfulness, fluid intelligence and resting state network resilience and integration.

## Methods

### Participants

Forty-seven participants, 16 yoga practitioners, 16 meditation practitioners, and 15 controls were recruited. Groups were matched for age, gender, education, race, and handedness. All participants self-reported being physically and mentally healthy. Individuals with current or past neurological conditions and individuals taking any medication for psychiatric or neurological conditions were excluded. Participants were also free of dementia as assessed with the Mini-Mental State Examination (Folstein et al., 1975). Yoga practitioners were trained in the Kripalu Yoga tradition (Faulds, 2005) and had an average of 13,534 (*SD* = 9, 950) hours of yoga experience. Meditators were trained in insight meditation (Goldstein and Kornfield, 2001) and had an average of 7, 458 (*SD* = 5, 734) hours of meditation experience. Controls had no experience with yoga or meditation. Participants were compensated with $100 and provided written informed consent. The study was approved by the Partners Human Research Committee, Massachusetts General Hospital (protocol 2005P001392).

### Behavioral measures

*Fluid intelligence*, comprising a variety of cognitive skills, was measured with the odd items of the Raven's Advanced Progressive Matrices (APM) (Raven et al., 1998; Raven, 2000). *Verbal intelligence*, which is a form of *crystallized intelligence* (Deary et al., 2010), was assessed with the American version of the National Adult Reading Test (AMNART; Grober and Sliwinski, 1991), *cognitive functioning* was assessed with the Mini-Mental State Examination (Folstein et al., 1975), and *mindfulness* with the Five Facet Mindfulness Questionnaire (FFMQ; Baer et al., 2006). To simplify analyses the sum score of the 5 subscales has been used, following the example of Carmody and Baer (2008). Furthermore, it was recorded for how many hours per week participants engaged in *physical exercise*, and *cognitive activities* including reading, writing, solving puzzles, and playing board and card games.

### Image acquisition

Imaging data was collected on a Siemens 1.5 Tesla Avanto MRI scanner (Erlagen, Germany) at the Martinos Center for Biomedical Imaging. Structural images were acquired using a T1-weighted magnetization prepared rapid acquisition gradient echo (MPRAGE) sequence (128 sagittal slices, slice thickness = 1.33 mm, TR = 2.73 s, TE = 3.39 ms, flip angle = 7°, field of view = 256 × 256 mm^2^, matrix = 192 × 192 mm^2^). A 5 min functional resting state scan was acquired using a gradient echo T2^*^-weighted sequence (TR = 2.5 s, TE = 40 ms, flip angle = 90°, field of view = 320 mm, matrix = 64 × 64 mm^2^). Twenty two horizontal slices with 1 mm gap, parallel to the inter-commissural plane (voxel size: 3.13 × 3.13 × 5 mm) were acquired inter-leaved.

### Procedure and analysis

Though it is expected that fluid intelligence declines with age, the model of this decline is not known a priori. A simple linear regression model would predict that fluid intelligence reaches negative values after a certain age, which is not consistent with the positive Raven's score. We therefore investigated different regression models which can be summarized by the general relation

(1) f(F)=A×g(age)+B

in which F is fluid intelligence measured by the Raven's score and f and g are two functions. We limited the analysis to f and g being either the identity, the logarithm or the inverse. These functions bring flexibility in the scale and proportionality of the relation between age and intelligence. Furthermore, they are all strictly increasing or decreasing functions, which is necessary to interpret A and B in terms of a decline. To select which of these models best represent the data, a model selection based on the Akaike information criterion (AIC) was carried out in the control group, the same way Dosenbach et al. (2010) selected their model of brain maturity. The parameter A of the regression indicates the rate at which intelligence declines with age. The parameter B, the generalized intercept, provides insight into the absolute value of the fluid intelligence. Group differences in parameters A and B were tested with permutation tests with 10,000 permutations. Permutation tests were one-sided except for the comparisons between yoga practitioners and meditators. Curve fitting and parameter comparisons were conducted with Matlab R2009a (The MathWorks Inc. Natick, MA, USA). This method was also used to investigate the relationship between age and the graph measures.

Relationships between mindfulness and other variables were tested with Pearson product-moment correlations. ANOVAs were conducted to compare the three groups in different variables. Significant ANOVAs were followed up by independent samples *t*-tests (two-tailed) to compare groups pair-wise. Whenever the assumption of homogeneity of variances was not met for ANOVAs, Welch's test of equality of means (Welch, 1951) was used. These analyses were done with SPSS 17 (SPSS Inc., Chicago, IL, USA).

#### Imaging

***Data preprocessing.*** Resting state data was slice time corrected, realigned, coregistered to individual T1-weighted images, normalized, and spatially smoothed with a 5 mm kernel using SPM8 (Wellcome Department of Cognitive Neurology, London, UK; www.fil.ion.ucl.ac.uk/spm/). Next, the first 8 image acquisitions of the resting functional time series were discarded to allow for stabilization of the MR signal. The remaining 112 volumes were further preprocessed using the Connectivity toolbox (http://www.nitrc.org/projects/conn; Whitfield-Gabrieli et al., 2011). Mean white matter signal, mean CSF signal, 6 motion parameters, and the first order motion derivative were regressed out of the data. Finally, the residual time series were band-pass filtered with a window of 0.008–0.09 Hz.

***Anatomical parcellation and timeseries extraction.*** Resting state scans were parcellated into 116 regions of interest (ROIs; 90 cortical and subcortical, and 26 cerebellar) using the Automated Anatomical Labeling (AAL; Tzourio-Mazoyer et al., 2002) template in the Wake Forest University (WFU) Pickatlas version 2.5 (Maldjian et al., 2003).

For each ROI, the preprocessed time-series was extracted, resulting in a 116 (ROIs) × 112 (volumes) time-series matrix for each subject. These matrices were used to generate bivariate correlation matrices for each subject. Time-series extraction was done with the Connectivity toolbox.

***Network analysis.*** Graph theory has recently been introduced in neuroscience to characterize the brain network (Bullmore and Sporns, 2009). In graph theory, networks are represented as a set of nodes connected by edges. Edges can be either weighted (to encode the strength of the connection) or unweighted. The weights can then be used to define a network distance that increases when the connection strength decreases. A path in a graph is a set of edges through which two distant nodes are connected. When different paths connect two nodes, the shortest path is the one with the smallest network distance. In this study, graphs were built with the 116 ROIs as nodes and the correlation between their mean BOLD time series as edge weights. To capture more of the richness of the available data, we used weighted graphs (Newman, 2004; Saramäki et al., 2007). Negative correlations were thresholded out. The functional distance was computed as the inverse of the correlation (Rubinov and Sporns, 2010).

To analyze the functional networks in terms of their integration and segregation, four graph measures were computed: characteristic path length, global efficiency, clustering coefficient and small-worldness. The mathematical definition of these measures can be found in Rubinov and Sporns (2010). Here, we summarize their use and interpretation. The characteristic path length is the average of the shortest path length between all pairs of nodes. This measure is primarily driven by longer paths. In particular, if two nodes are disconnected, their shortest path will be infinite, and so will be the characteristic path length. The latter is therefore a measure of integration of functionally distant brain regions. The global efficiency is the average of the inverse of the path length between all pairs of nodes. This measure is mostly driven by shorter paths. It is therefore a measure of integration between functionally close brain regions. The clustering coefficient measures how likely two nodes that are strongly connected to a third one are also strongly connected to each other, forming a strongly connected triangle in the network. As such, it is a measure of segregation, which is the ability to perform specialized tasks in a densely connected network. These three measures were normalized by their mean over 500 surrogate networks created by randomly shuffling the edges. This normalization eliminates the potential effect of a global difference between connection strengths in the different groups. A fourth measure, small-worldness, can be computed as the ratio between the normalized clustering coefficient and the characteristic path length. Small-world property of a network defines how well the network finds a trade-off between a highly integrated network (as seen in random graphs) and a highly segregated network (as seen in a regular lattice).

***Resilience.*** Functional networks can also be characterized by their resilience to attacks (Achard et al., 2006). Whether attacks represent actual lesions in the cortex or simulate the normal degenerescence of aging neural units, a resilient brain network will be less affected by them. In practice, attacks to the functional networks are simulated by removing nodes and computing some network property of the resulting damaged network. Two types of attacks are considered: random failures where nodes are removed randomly and targeted attacks where the removed node is chosen based on its degree centrality. Degree centrality refers to how important the node is in facilitating functional integration and is defined as the sum of the weights of all edges that connect to the node (Rubinov and Sporns, 2010). Physiologically, targeted attacks may better simulate neurodegenerescence, as highly connected nodes tend to be more metabolically active, making them more vulnerable (Alstott et al., 2009). The property computed after each attack can be the characteristic path length or the global efficiency (Albert et al., 2000; Achard et al., 2006). However, global efficiency is usually preferred since it can be computed even if the network is disconnected, while the characteristic path length goes to infinity in that case (Rubinov and Sporns, 2010). Here we reported statistical group comparisons of resilience to targeted network damage of 15 nodes (i.e., the 15 nodes with the greatest degree centrality were removed) in terms of global efficiency. However, results were similar for the other sizes of attacks (3–30 nodes in steps of 3). All network measures were computed using NetworkX, a set of open-source tools written in Python (Hagberg et al., 2008).

## Results

### Subject characteristics

Importantly for this study, yoga practitioners, meditators, and controls were matched for age, gender, education, race, and handedness (Table 1). Groups also did not significantly differ in the potentially confounding variables verbal intelligence, current number of hours spent exercising, and current number of hours spent engaged in cognitive activities such as reading, writing, and board and card game playing (Table 1).

**Table 1:** **Group comparisons of matching variables and other potential confounders**.

	**Controls**		**Yoga practitioners**		**Meditators**		**ANOVA/χ^2^-test**		
	***M/*%**	***SD***	***M/*%**	***SD***	***M/*%**	***SD***	***F/*χ^2^**	***df***	***p***
Age (years)	52.93	9.84	49.38	7.79	54.06	8.15	1.29	2, 44	0.286
Education (years)	17.27	1.98	17.31	2.41	18.44	2.58	1.26	2, 44	0.293
AMNART VIQ	120.73	7.65	123.27	4.67	125.60	4.70	2.34^a^	2, 27	0.115
Exercise (h/week)	4.79	4.53	6.98	5.57	6.48	4.16	0.72	2, 36	0.496
Read/Write/Play (h/week)	23.75	8.89	21.07	15.76	13.27	11.41	2.13	2, 34	0.134
Gender (% female)	60%		69%		63%		0.28	2	0.871
Handedness (% right)	87%		88%		88%		0.01	2	0.997
Race (% white)	100%		100%		100%				

a Welch test. AMNART, American National Reading Test.

### Age related decline in fluid intelligence

**Figure 1:**
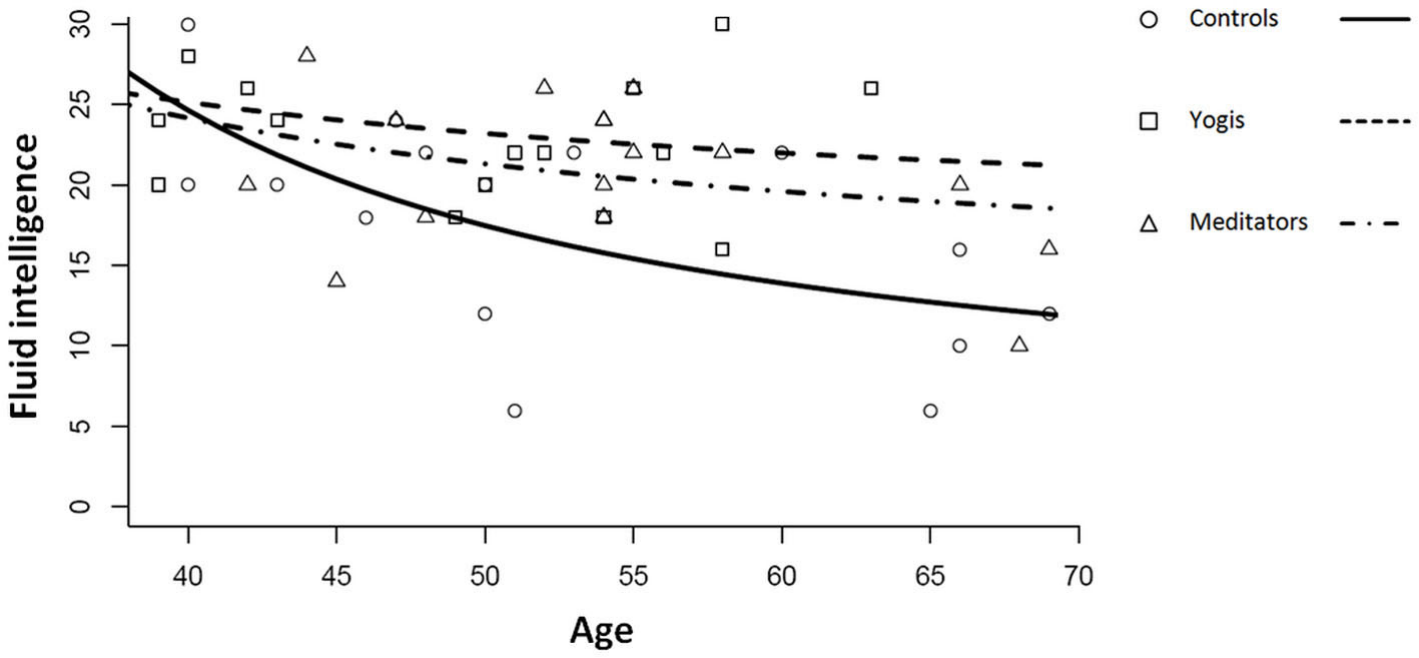
**Relation between age, measured in years and fluid intelligence measured with the Raven's Advanced Progressive Matrices for controls, yoga practitioners, and meditators**. Lines represent log-linear regression lines for the three groups following the function log(F)=A/age+log(F_f_).

The best fitting model for the relation between age and the APM score was a log-linear model including the inverse age (Equation 2; Figure 1).

(2) $$\text{log}(\text{F})=\text{A/age}+\text{log}({\text{F}}_{\text{f}}).$$

Where A corresponds to the rate at which fluid intelligence declines, and F_f_ expresses the plateau that fluid intelligence reaches at advanced ages.

Coefficient A was significantly lower for yoga practitioners and meditators considered as a single group than for controls (permutation test; *p* = 0.040). The difference was mostly driven by the difference between yoga practitioners and controls (permutation test; *p* = 0.057) and only slightly by the difference between meditators and controls (permutation test; *p* = 0.092). There was no significant difference in decline rate between yoga practitioners and meditators (*p* = 0.190). In this model, the intercept B = log(F_f_) indicates the final plateau value that is reached at advanced ages. It is another characteristic of the decline profile and we can therefore expect it to be larger for yoga practitioners and meditators. *Post-hoc* permutation tests revealed that this parameter was significantly higher in yoga practitioners and meditators considered as a single group as compared to controls (*p* = 0.019). This difference was mainly driven by the difference between yoga practitioners and controls (*p* = 0.032) and only in part by the difference between meditators and controls (*p* = 0.070). There was no significant difference between yoga practitioners and meditators (*p* = 0.140).

Possibly as a result of the slower decline of fluid intelligence in yoga practitioners and meditators as compared to controls, we found a significant main effect for group [*F*_(2, 44)_ = 4.827, *p* = 0.013, η^2^_*p*_ = 0.180] in a one-way ANOVA with group (controls, meditators, yoga practitioners) as factor, and Raven's APM score as dependent variable. *Post-hoc t*-tests revealed that yoga practitioners (*M* = 23.500, *SD* = 4.761) had significantly higher Raven's APM scores than controls [*M* = 17.333, *SD* = 6873; *t*_(29)_ = 2.920, *p* = 0.007], while their scores did not significantly differ from those of meditators [*M* = 20.750, *SD* = 4.782; *t*_(30)_ = 1.630, *p* = 0.114]. Meditators and controls did not significantly differ in Raven's APM scores [*t*_(29)_ = 1.615, *p* = 0.117]. When considered as a single group, yoga practitioners and meditators had significantly higher Raven's APM scores than controls [*t*_(45)_ = 2.741, *p* = 0.009].

Together, these findings are consistent with the hypothesis that yoga practitioners and meditators combined had a lower rate of decline of fluid intelligence than controls, which was mostly driven by a reduced decline in yoga practitioners. This apparent reduced decline might be the cause for the greater fluid intelligence that we observed in our combined group of older yoga practitioners and meditators. Furthermore, our model predicts that fluid intelligence will not decline beyond a certain level and that this is higher for yoga practitioners and meditators than for controls.

### Integration and segregation of resting state networks

**Figure 2:**
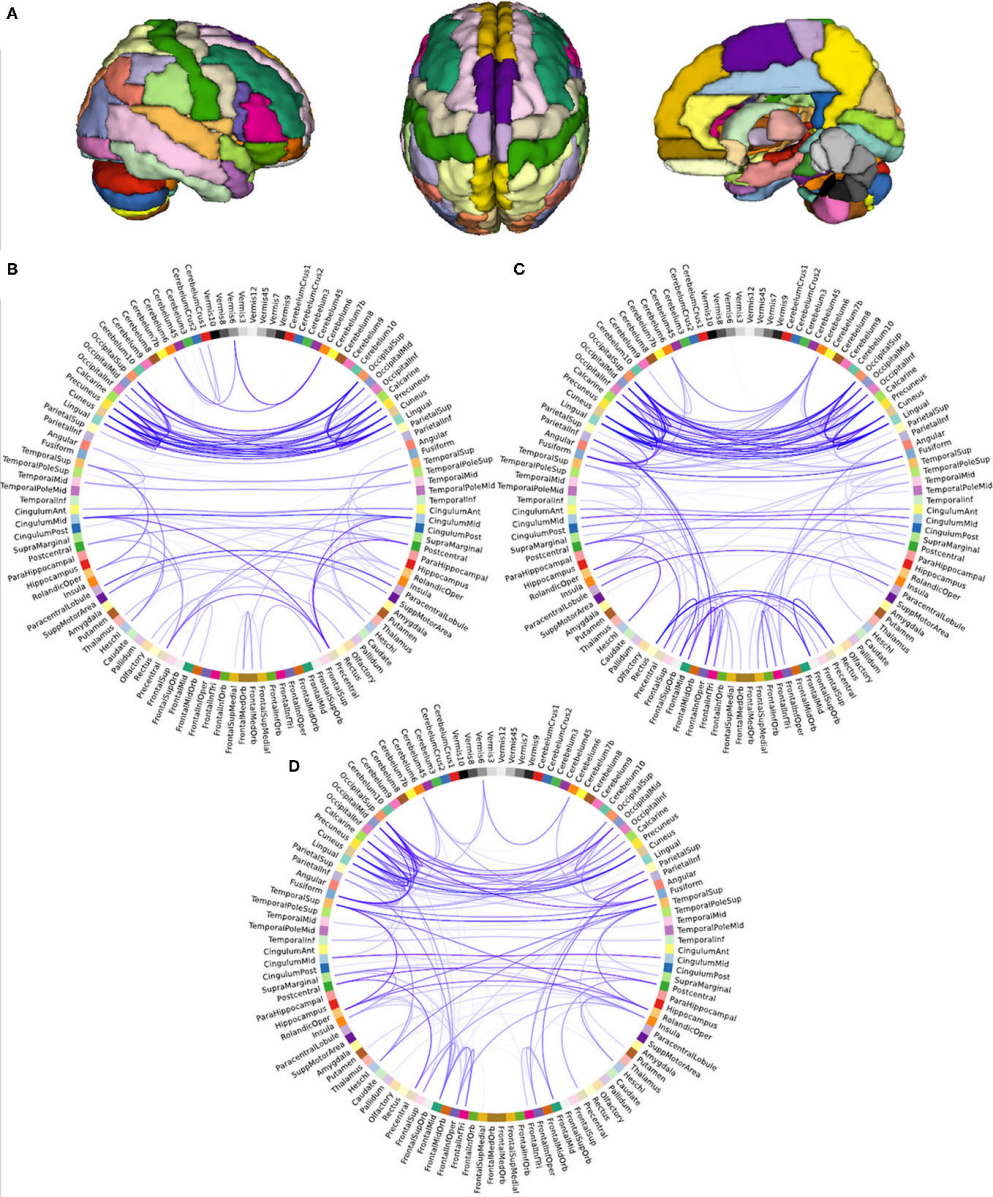
**Visualization of network creation**. Parcellation of brain into 116 regions **(A)**, and structure of weighted resting state functional networks of a representative control **(B)**, yoga practitioner **(C)**, and meditator **(D)**. Networks depicted were thresholded (*r* = 0.5) for illustrative purposes only. Opacity of the lines represents strength of the correlation (more saturated is stronger), relative to the strength of the strongest correlation.

**Figure 3:**
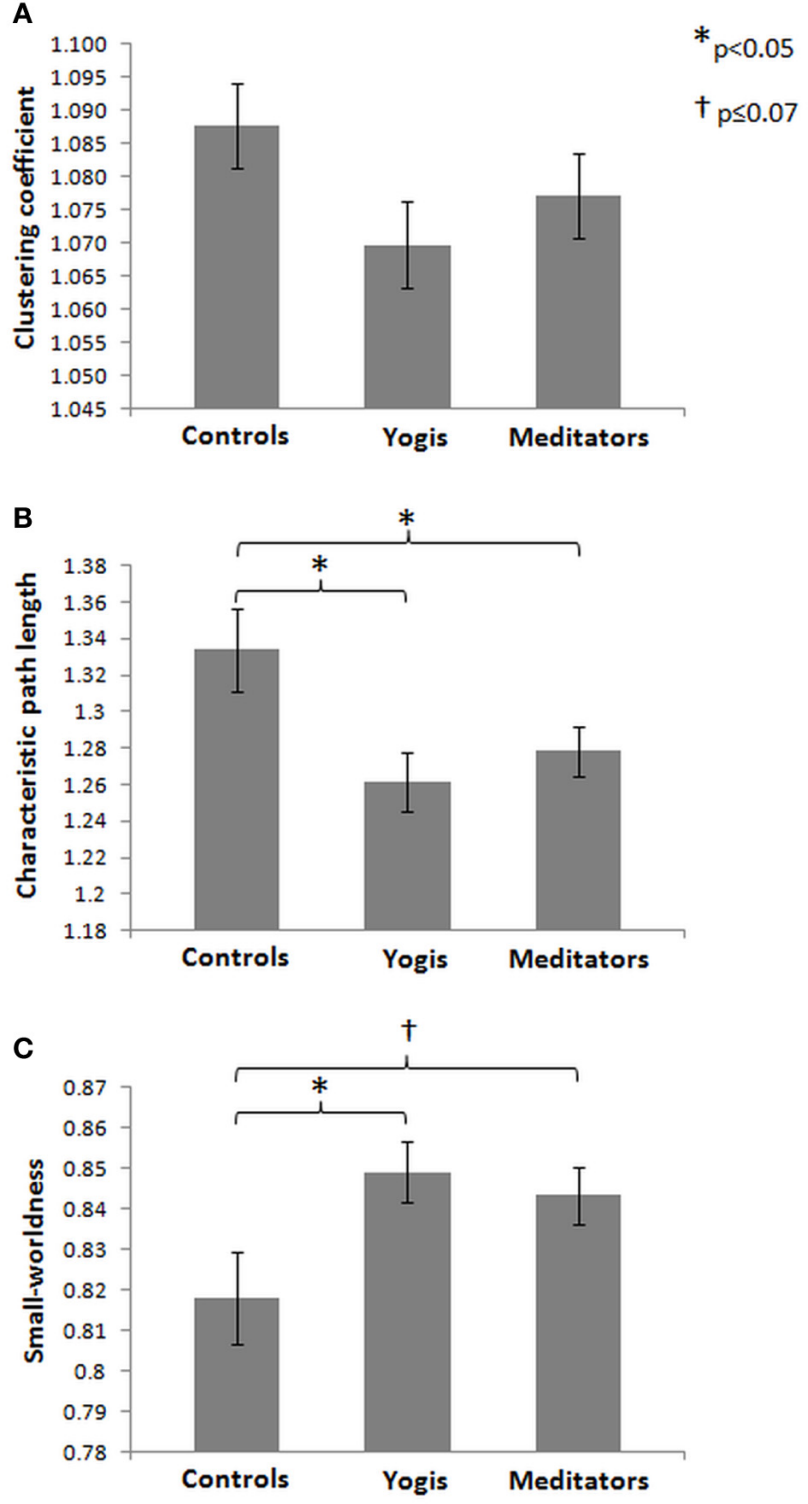
**Mean of normalized clustering coefficient (A), normalized characteristic path length (B), and small-worldness (C) for controls, yoga practitioners, and meditators, based on weighted resting state brain functional networks**. Error bars represent the standard error of the mean. *P*-values are based on two-tailed independent samples *t*-tests.

To compare the brain functional network architecture of yoga practitioners, meditators and controls (for a visualization of the network creation see Figure 2) in terms of small-worldness, characteristic path length, global efficiency and clustering coefficient, ANOVAs were conducted. Significant group differences were found in normalized characteristic path length [*F*_(2, 44)_ = 4.482, *p* = 0.017, η^2^_*p*_ = 0.169; Figure 3B] and normalized small-worldness [*F*_(2, 44)_ = 3.487, *p* = 0.039, η^2^_*p*_ = 0.137; Figure 3C], and a trend toward significance in normalized global efficiency [*F*_(2, 44)_ = 3.148, *p* = 0.053, η^2^_*p*_ = 0.125]. However, there were no significant group differences in the normalized clustering coefficient [*F*_(2, 44)_ = 1.956, *p* = 0.153, n.s.; Figure 3A]. *Post-hoc* independent samples *t*-tests revealed that yoga practitioners and meditators each had significantly shorter characteristic path length than controls [*t*_(29)_ = −2.652, *p* = 0.014 and *t*_(23.34) = −2.100_, *p* = 0.047, respectively]. This was also true when considered as a single group [*t*_(20.33)_ = −2.562, *p* = 0.018]. There was no difference between yoga practitioners and meditators [*t*_(30) = −0.782_, *p* = 0.440; Figure 3B]. Yoga practitioners also had significantly greater global efficiency than controls [*t*_(29)_ = 2.249, *p* = 0.032], while the difference between meditators and controls showed a trend toward significance [*t*_(29)_ = 1.893, *p* = 0.068]. Combined, yoga practitioners and meditators had significantly greater global efficiency than controls [*t*_(45)_ = 2.428, *p* = 0.019], while there was no significant difference between yoga practitioners and meditators [*t*_(30)_ = −0.701, *p* = 0.489]. Furthermore, *post-hoc* independent samples *t*-tests revealed that yoga practitioners had significantly greater small-worldness than controls [*t*_(29)_ = 2.297, *p* = 0.029]. Meditators had borderline significant greater small-worldness than controls [*t*_(23.60)_ = 1.899, *p* = 0.070]. Yoga practitioners and meditators together had significantly greater small-worldness than controls [*t*_(19.84)_ = 2.261, *p* = 0.035], while there was no difference between yoga practitioners and meditators [*t*_(30)_ = 0.535, *p* = 0.596; Figure 3C].

Attempts to find the best fitting function for the relationship between age and each of the graph measures revealed that although all models had poor fit, the linear regression model was the best fitting model (all *p* > 0.050). None of the slopes or intercepts differed significantly between any of the groups for any of the graph measures (all *p* > 0.050). These findings indicate that although no group differences in age-related decline were found in network integration or segregation, yoga practitioners and meditators combined had greater network integration but not segregation than controls. Again, this difference was mostly driven by the yoga practitioners.

### Resilience of resting state networks

**Figure 4:**
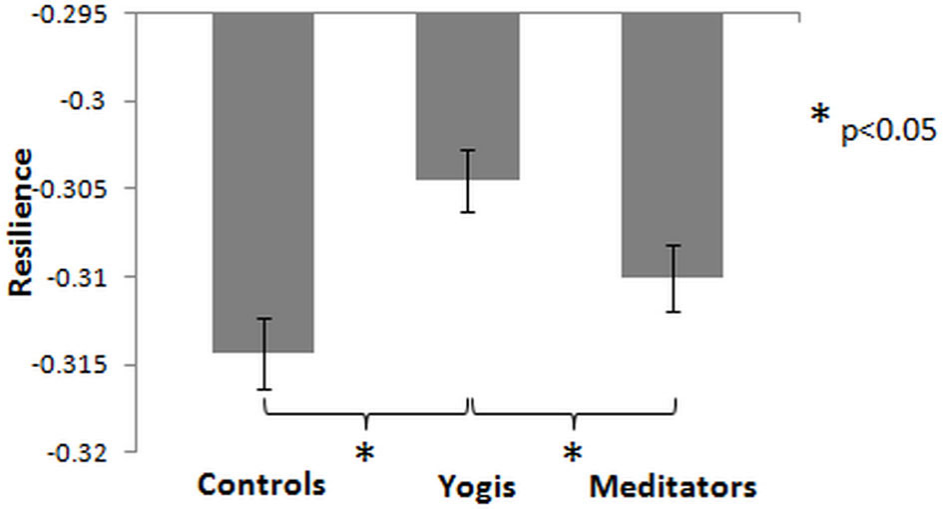
**Mean of functional network resilience, measured as the change in global efficiency after removal of the 15 nodes with the highest degree centrality**. Less decrease means higher resilience. Error bars represent the standard error of the mean. *P*-values are based on two-tailed independent samples *t*-tests.

We assessed the resilience of the functional networks to targeted damage (Achard et al., 2006), by removing nodes from the networks in the order of their degree centrality. An ANOVA with group as factor and change in normalized global efficiency after removal of 15 nodes as dependent variable, revealed a significant group effect [*F*_(2, 44)_ = 6.979, *p* = 0.002, η^2^_*p*_ = 0.241; Figure 4]. *Post-hoc* independent samples *t*-tests revealed that this group difference was driven by a smaller decrease in yoga practitioners than in controls [*t*_(29)_ = 3.749, *p* = 0.001] and meditators [*t*_(30)_ = 2.161, *p* = 0.039], and no significant difference was found between meditators and controls [*t*_(29)_ = 1.594, *p* = 0.122]. When yoga practitioners and meditators were considered as a single group, attacks resulted in a significantly smaller decrease in global efficiency than in controls [*t*_(45)_ = 2.964, *p* = 0.005]. These findings indicate that considered as one group, yoga practitioners and meditators have more resilient networks than controls, but when considered separately, yoga practitioners have more resilient networks than controls and meditators.

### Mindfulness, intelligence, and network integration

**Figure 5:**
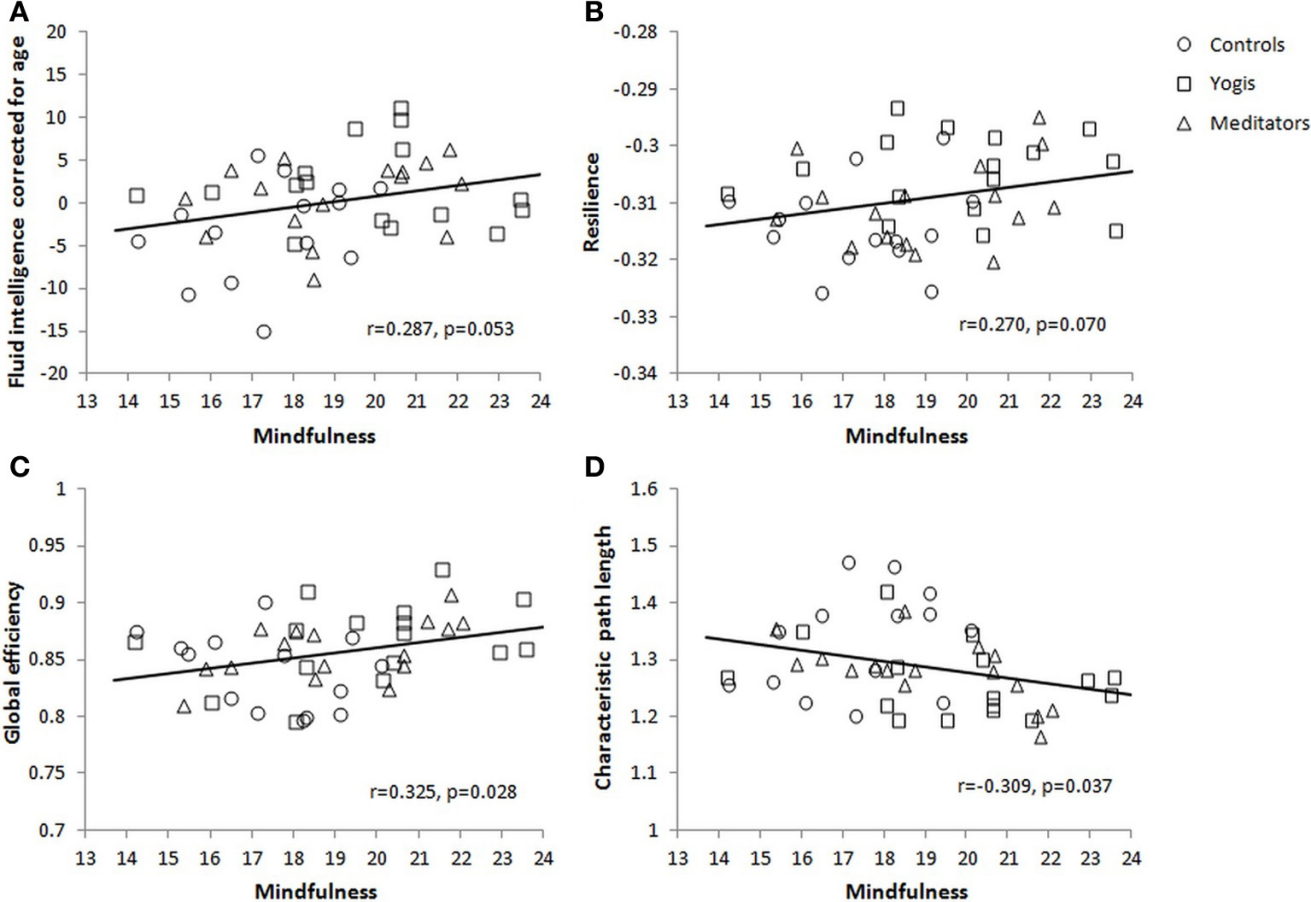
**Correlations between mindfulness and (A) fluid intelligence as measured with the Raven's Advanced Progressive Matrices and corrected for age, (B) brain functional network resilience for targeted damage of 15 nodes, (C) global efficiency, and (D) characteristic path length**.

The three groups differed in sum score of the FFMQ [*F*_(2, 44)_ = 5.544, *p* = 0.007, η^2^_*p*_ = 0.201]. This group difference was driven by significantly greater scores in yoga practitioners [*M* = 19.780, *SD* = 2.576; *t*_(29)_ = 3.006, *p* = 0.005] and meditators [*M* = 19.053, *SD* = 2.190; *t*_(29)_ = 2.426, *p* = 0.022] than in controls (*M* = 16.826, *SD* = 2.894). There was no significant difference between yoga practitioners and meditators [*t*_(30)_ = 0.860, *p* = 0.396]. To investigate the relationship between mindfulness and fluid intelligence, the correlation between the sum score of the FFMQ and the age corrected sum score of the Raven's APM was calculated. This correlation was significant [*r*_(45)_ = 0.292, *p* = 0.046] and still approached significance after removing an outlier on mindfulness [*r*_(44)_ = 0.287, *p* = 0.053; Figure 5A]. Mindfulness was also significantly correlated with network resilience as defined in the previous section [*r*_(45)_ = 0.329, *p* = 0.024], and still approached significance after removing an outlier on mindfulness [*r*_(44)_ = 0.270, *p* = 0.070; Figure 5B]. Furthermore, mindfulness was positively related to network integration as reflected in a positive correlation of mindfulness with normalized global efficiency [*r*_(45)_ = 0.367, *p* = 0.011] and a negative correlation with normalized characteristic path length [*r*_(45)_ = −0.356, *p* = 0.014]. Both correlations remained significant after removing an outlier on mindfulness [*r*_(44)_ = 0.325, *p* = 0.028; Figure 5C, and *r*_(44)_ = −0.309, *p* = 0.037; Figure 5D, respectively]. Together these findings indicate that individuals who are more mindful have higher fluid intelligence and more integrated and resilient resting state brain networks.

## Discussion

In this study we investigated age-related decline in fluid intelligence and resting state functional brain network properties in older yoga practitioners, meditators, and controls. Importantly, the groups were well-matched demographically and did not significantly differ in several factors that impact brain function, including age, gender, education, crystallized intelligence, and average hours of physical exercise and cognitive engagement in daily life. Aligned with our hypothesis, the apparent rate of age-related decline in fluid intelligence was lower in yoga practitioners and meditators combined as compared to controls. Congruent with this finding, resting state functional brain networks of yoga practitioners and meditators combined were more resilient to damage than those of controls and had a stronger small-world architecture. This difference in small-worldness was driven by greater network integration rather than segregation. Interestingly, mindfulness was positively related to fluid intelligence as well as network resilience and integration. These findings link behavioral training to reduced decline in fluid intelligence and greater functional brain network integration and robustness in older adults. In addition, the correlation between mindfulness and network integration, resilience, and fluid intelligence suggests that mindfulness plays a mechanistic role in this preservation and advances our understanding of the involved constructs.

### Slower decline of fluid intelligence

Consistent with our hypothesis of off-set age-related decline in yoga and meditation practitioners, decline parameters of the function describing the relationship between age and fluid intelligence were smaller in yoga practitioners and meditators combined than in controls. Presumably because of this apparent slower decline, the group averages of fluid intelligence were higher in the combined group of yoga practitioners and meditators than in the age-matched controls. These findings are consistent with previous studies which indicated that older meditators have better attentional performance than non-meditators (Van Leeuwen et al., 2009; Prakash et al., 2012), and that meditators don't show the normal negative correlation between sustained attention and age (Pagnoni and Cekic, 2007). Our results extend these earlier findings by revealing a reduced cognitive aging effect in yoga practitioners and meditators on the higher level construct fluid intelligence. This extension is important as fluid intelligence is a broader construct than attention and predicts meaningful behavior in the real world (Sternberg, 2008; Deary et al., 2010).

The question that these findings raise is what variables mediate the effect of meditation and yoga on fluid intelligence? The positive correlation between mindfulness and fluid intelligence we reported here suggests that mindfulness is one of these variables. Our finding is congruent with previous studies that have shown that both mindfulness (Moore and Malinowski, 2009; Anicha et al., 2012) and fluid intelligence (Colzato et al., 2006) are related to cognitive flexibility, thereby linking the two constructs.

### Resilient brain functional networks

Resilience can be studied in terms of change in brain network properties after simulated aging-related brain damage. Our analysis revealed that resting state functional brain networks of yoga practitioners and meditators together had greater resilience to simulated damage than those of controls. This finding is aligned with our finding of greater resilience to age-related cognitive decline. While decreased network resilience of structural brain networks has been reported in Alzheimer's patients (He et al., 2008), our data reveal conditions that may enhance network resilience. Although it is tempting to speculate that these network findings might translate to biological brain resilience, this link has not yet been established.

### Greater small-worldness through more integration

Recent studies have shown that experienced meditators have altered resting state functional connectivity within the default mode network (Jang et al., 2011; Taylor et al., 2012), and increased connectivity between the posterior cingulate and the dorsal anterior cingulate and dorsolateral prefrontal cortices (Brewer et al., 2011). The current study extends these findings by demonstrating differences in graph theoretical global network properties, thereby capturing more of the full complexity of the functional network architecture.

We found between-group differences in small-worldness, characteristic path length, and global efficiency, but not in clustering coefficient. Yoga practitioners and meditators combined had significantly shorter characteristic path length and greater global efficiency and greater small-worldness than controls. Elevated small-worldness of the yoga practitioners and meditators was driven by higher network integration (characteristic path length and global efficiency), rather than by increased network segregation (clustering coefficient). Hence, aging yoga practitioners and meditators have a more small-world like resting state functional brain network architecture compared to controls by having stronger integrative connections, efficiently integrating information from distributed brain regions, rather than having more groups of highly connected local brain regions for specialized information processing.

Long distance γ-synchrony has long been hypothesized to be a mechanism for large scale network integration related to coherent cognition, behavior, and consciousness (Singer, 1993; Rodriguez et al., 1999; Varela et al., 2001), and a recent study revealed that long-distance synchronization between brain regions, especially in the β (16–32 Hz) and γ (32–63 Hz) bands is related to cognition and network integration as measured by global efficiency (Kitzbichler et al., 2011). Interestingly, increased long-distance gamma (25–42 Hz) synchrony has been related to meditation, with a significant group (meditators vs. controls) by condition (meditation vs. baseline) interaction (Lutz et al., 2004). Our finding of shorter characteristic path length in yoga and meditation practitioners than in controls extends this finding by revealing group differences in network integration during rest and by demonstrating altered network integration in yoga practitioners.

We did not find a significant decline of any of the network properties with age, and no differences in decline between groups. The absence of a decline in clustering coefficient is in agreement with a previous study that did not find a difference in modularity, another measure of network segregation, between young and old healthy subjects (Meunier et al., 2009).

Although we did not find the expected negative correlation between age and network integration, probably due to our relatively small sample size and the relatively narrow age range (39–69 years), growing evidence suggests that there is an age related decline of integration in functional networks (Achard and Bullmore, 2007). Achard and Bullmore (2007) found that young (*M* = 24.7 years) participants had greater global efficiency than old (*M* = 66.5 years) participants. Thus, with a more integrated, more efficient network organization as compared to controls, yoga practitioners and meditators might be viewed as having a younger, less-degraded brain network organization. These findings indicate that it might be possible to reduce age related decline in functional network integration through meditation and yoga practice.

### Mindfulness is related to fluid intelligence and brain functional network resilience and integration

The correlation between fluid intelligence and mindfulness that we report here is congruent with previous studies linking both variables to cognitive flexibility (Colzato et al., 2006; Moore and Malinowski, 2009; Anicha et al., 2012). We also found a positive correlation between mindfulness and global efficiency, as well as a negative correlation between mindfulness and characteristic path length. Together, these findings are aligned with previous reports of negative correlations between characteristic path length and fluid intelligence (Van Den Heuvel et al., 2009; Langer et al., 2012). Similarly, using diffusion tensor imaging (DTI) tractography derived networks, Li et al. (2009) reported a positive correlation between global efficiency and intelligence, and a negative relation between characteristic path length and intelligence. Wen et al. (2011) reported positive correlations between global efficiency and processing speed, visuospatial ability, and executive function. The correlation of mindfulness with measures of network resilience, network integration and fluid intelligence suggests that mindfulness plays a central role in the prevention of age related cognitive and neural decline.

### Yoga practitioners vs. meditators

To date, much of the neuroimaging literature regarding mind-body practices has focused on mindfulness meditation. However there are numerous yoga and meditation traditions, as well as other mind-body practices such as tai chi. Although there are important philosophical differences between different Eastern traditions, there are also numerous similarities (Goleman, 1996). Previous longitudinal studies that have directly compared different styles of meditation or yoga have reported both convergent and divergent findings. Wolever et al. (2012) failed to show a difference between a yoga-based and a mindfulness-based intervention on a number of psychological and physiological variables, though both practices showed greater improvements on perceived stress, sleep quality, and aspects of heart rate variability in a workplace setting than a control condition. However, other studies have found between-group differences between mindfulness and relaxation interventions on various scales and tests, including rumination (Jain et al., 2007), and emotional interference, psychological well-being, and negative affect (Ortner et al., 2007).

In the current study we recruited individuals whose main practice was either Kripalu yoga or Vipassana (a.k.a. mindfulness) meditation. Mindfulness plays a central role in both of these traditions (Carmody and Baer, 2008; Nyklicek and Kuijpers, 2008; Shelov et al., 2009; Gard et al., 2012; Wolever et al., 2012), though in Kripalu the concept of mindfulness is referred to as “witness consciousness” (Faulds, 2005); and see (Gard et al., 2012) for a comparison of these concepts. The two groups did not differ significantly on any of our metrics, except for network resilience which suggests these practices have similar effects on brain network organization and cognitive functioning. The significantly greater resilience in yoga practitioners than in meditators and the overall trend for larger differences between yoga practitioners and controls with meditators in between likely due to the fact that the yoga group had almost twice as much life-time hours of practice as the meditation group.

### Limitations

It is important to note that a variety of factors are thought to influence the rate of normal age-related decreases in brain structure and function including gender, cognitive engagement and physical exercise (Plassman et al., 2010; Fotuhi et al., 2012; Wilson et al., 2012). Therefore we matched yoga practitioners, meditators and controls for age, gender, race, handedness, and education, and additionally compared them on verbal intelligence, current exercise, and cognitive spare time engagement (reading, writing, playing games). Although groups did not significantly differ on any of these variables, there might be other confounding variables that we did not control for.

Since the presented study has a cross-sectional design it has important limitations, such that causality cannot be established. For example, people endowed with higher fluid intelligence and greater brain functional network integration may be more interested in practicing yoga and meditation. While our present study with highly experienced yoga practitioners and meditators revealed interesting and encouraging findings, future longitudinal studies with larger sample sizes and individuals without prior experience with these mind-body techniques will be required to establish causality.

## Conclusion

We have provided evidence that is in line with the hypothesis that age-related decline in fluid intelligence is slower in yoga practitioners and meditators and that these practitioners have more efficient and resilient functional brain networks than matched controls. Furthermore, we reported no significant differences between yoga practitioners and meditators on most of the assessed variables and found that mindfulness, which is a key skill developed through meditation and yoga practice, was positively correlated with fluid intelligence and global brain network efficiency and resilience. These findings are of theoretical importance as they provide insight into the global brain functional network architecture of yoga practitioners and meditators and provide a potential mechanism for the preserved intellectual capacity in mindfulness practitioners. Furthermore, these findings have potential practical implications with a rapidly aging world population and increasing life expectancies (United Nations, 2002; Administration on Aging, 2012). Longitudinal research is needed to establish causality between the practice of yoga and meditation and reduced decline of intelligence and functional brain network architecture.

### Conflict of interest statement

This study was in part funded by the Kripalu Center for Yoga and Health. As part of the study involved the investigation of yoga practitioners trained in the tradition of Kripalu yoga, there might be a perceived conflict of interest. However, the Kripalu Center for Yoga and Health was not involved in the design, conduct, analysis, or reporting of the study. The authors declare that the research was conducted in the absence of any commercial or financial relationships that could be construed as a potential conflict of interest.
